# Basic occupational health services

**DOI:** 10.4103/0019-5278.50715

**Published:** 2009-04

**Authors:** Shyam Pingle

**Affiliations:** Reliance Industries Ltd., Mumbai, India. President, Indian Association of Occupational, Health. Email: Shyam.Pingle@ril.com

The Constitution of India states that ‘State shall make provisions for securing just and humane conditions of work’. This provides the basis for provision of occupational health services to all citizens of the country.

However, in reality, there is plenty of opportunity to provide occupational health services to all working population, not only in India, but even in the developed world. Occupational health services are available only to 10-15% of workers worldwide and to a miniscule of working population in developing countries. Even where services are available, the quality and relevance may be low. Though there is an intense economic pressure on cost of production all over the world, there cannot be a trade-off between health and productivity at work.

The Basic Occupational Health Services (BOHS) are an application of the primary health care principles in the occupational health sector. The BOHS seek to provide occupational health services for all working people in the world regardless of mode of employment, size of workplace or geographic location, that is, according to the principle of universal services provision. These services are most needed in countries and sectors which do not have services at all or which are seriously underserved. It lays stress on the importance of a national strategy and plan of action to incorporate occupational health in all policies.

The concept of BOHS has been developed jointly by the World Health Organization (WHO), International Labor Organization (ILO), and International Commission on Occupational Health (ICOH) and has its roots in the ‘Alma Ata’ declaration (1978) by the WHO. The BOHS principles were first discussed at the WHO/ILO Joint Committee of Occupational Health in 2003. The BOHS has become a central piece of global occupational health services development plans of the WHO and ILO. The WHO, with its collaborating centers in occupational health, the ILO, ICOH and other international organizations, work for the BOHS. The BOHS shot into limelight with outgoing ICOH President, Prof. Jorma Rantanen, championing the cause.

The BOHS concept envisages coverage of all workers, and has a strong focus on prevention. They are to be provided for SMEs as well as self employed persons through public services. There will have to be different modalities for the same. There has to be a strong primary health care approach, which needs strong coordination between health and labor ministries, in our country. The expert institutions on occupational health have an important role to play in BOHS and they need to support the provision of BOHS by developing low-cost solutions.

The BOHS aim at:

- Protection of health at work,
- Promotion of health, well being, work ability and
- Prevention of occupational diseases and accidents.

Activities under BOHS encompass not only health surveillance, emergency preparedness and first aid services but also include surveillance of work environment, risk assessment and preventive and control measures. Health education and health promotion are also an integral part of BOHS.

The BOHS provide a practical tool in identifying priorities and pooling scarce resources to develop an integrated and effective occupational health system and services, tailored to suit the national conditions and needs of each country. Improved conditions of work will lead to a healthier work force and, in turn, improved productivity.

It is estimated that India has a working population of approximately 500 million. According to 2001 census, around 70% of the population resides in rural areas. Less than 10% of the workforce is organized, 60% self-employed and 30% do not have regular jobs. The increasing proportion of females in the workforce adds to the traditional OSH issues. The changing face of service sector, in view of the exponential growth on account of globalization and increasing use of information technology, is expected to present new challenges.

Proper diagnosis and reporting of occupational diseases is necessary to achieve and implement BOHS. As all of us are aware, the statistics on accidents and occupational illnesses are far from accurate. There are research reports that show the official estimates are vastly low. The organized sector, both private and public, has reasonably well developed OHS based on ILO conventions. However, this sector is miniscule. The OHS are almost non-existent in the unorganized sector.

Currently, there is no government agency or department which deals exclusively with occupational safety and health matters. The director general of the Factory Advisory Services and Labor Institutes deals with the safety and health of workers employed in factories and ports, whereas, the director general of Mines Safety deals with the safety and health of miners. While there are other departments under the Ministry of Labour, which deal with OSH issues in different sectors, e.g. the construction sector, no agency covers safety and health for workers in unorganized sectors.

In India, we face the twin challenges of integration of occupational health with general health services and delivery of occupational health from medical college hospitals. There is separate training on occupational safety and health for safety professionals and occupational health professionals. The training on occupational health is still at an early stage and there are still no Chairs on occupational health in Indian universities and there are hardly any postgraduate training facilities on OH.

The BOHS demands government leadership with tripartite or better still, quadripartite collaboration between government, employers, employees and non-governmental organizations (NGOs) like IAOH. We need development of appropriate OSH infrastructure and proper dissemination of health and safety information. Our institutions need to provide simple tools for practical health and safety work at workplaces. Needless to add, our focus needs to be on small and medium sized enterprises, self-employed persons and informal sector.

Recently, the national occupational health and safety policy has been finalized by the government and let us hope that it will take the country one step closer towards BOHS for all.
